# Assessment of the Pennation Angle of the Medial Gastrocnemius Muscle in Road Runners and Non-runners

**DOI:** 10.1055/s-0045-1804493

**Published:** 2025-04-15

**Authors:** Lara Barros Cecílio Mendes, Carlos Henrique Fernandes, Paulo Santoro Bellangero, Benno Ejnisman, Moisés Cohen

**Affiliations:** 1 Disciplina de Medicina do Esporte e Atividade Física, Centro de Traumatologia do Esporte, Departamento de Ortopedia e Traumatologia, Escola Paulista de Medicina, Universidade Federal de São Paulo, São Paulo, SP, Brasil; 2 Departamento de Ortopedia e Traumatologia, Escola Paulista de Medicina, Universidade Federal de São Paulo, São Paulo, SP, Brasil

**Keywords:** motor activity, muscle contraction, muscle, skeletal, sedentary behavior, ultrasonography

## Abstract

**Objective** To evaluate the pennation angle (PA) of the medial gastrocnemius muscle (MGM) in orthostasis and bilateral maximum plantar flexion (BMPF) in road runners and non-runners.

**Methods** We assessed 31 runners and 31 non-runners from both genders between January and April 2019. We measured the MGM's PA on both sides by ultrasound during orthostasis and BMPF.

**Results** The groups were homogeneous regarding the dominant side, gender, and age. During orthostasis, the mean right-sided PA was 19.46° in runners and 22.5° in non-runners ( *p* < 0.004). On the left side, the mean PA was 20.79° in runners and 22.83° in non-runners ( *p* < 0.029). During BMPF, the right-side PA was 40.06° in runners and 40.89° in non-runners, and, on the left side, the mean PA was 40.01° in runners and 40.52° in non-runners. The MGM's PA of the right limb from male runners was statistically significantly higher in orthostasis ( *p* < 0.029) and BMPF ( *p* < 0.045). The correlation between MGM in orthostasis and BMPF in the leg in each group was statistically significant in the right lower limb of non-runners.

**Conclusion** Road runners presented a significantly lower MGM's PA than non-runners in orthostasis.

## Introduction

Running started in prehistoric times when humans needed to move quickly to hunt or escape predators. Running, even for 5 to 10 minutes per day and at slow speeds (< 9.65 km/h), is associated with significantly reduced risk of death from all causes and, specifically, from cardiovascular disease. The present study may motivate healthy but sedentary subjects to start and continue running to achieve substantial mortality reduction benefits. 1

Human muscle architecture has been studied in cadaveric specimens. However, scarce data are available on living human muscles. 2 3 4 Skeletal muscle tissue has properties that determine its characteristics and performance, such as excitability, contractility, extensibility, and elasticity. 5 The mechanical properties of skeletal muscle account for its performance, such as strength, length, speed, work, and power. 6

The pennation angle (PA) of the muscle fiber is the angle between the muscle fascicle and the intramuscular tendon, that is, the vertical inclination of the muscle fibers from the long muscle-tendon axis. 5 7 8 The fiber PA is a crucial functional feature of the muscle and varies according to the muscle contraction intensity and fiber length. 9 Muscle contraction causes fibers to shorten, increasing the PA. 9 10 Ultrasound allows accurate measurements of the length or PA of a muscle fascicle. 10 11 12

Muscle pain in the posterior region of the lower limb is a common complaint among runners. 13 14 Of all calf muscle injuries, 58 to 65% involve the medial gastrocnemius muscle (MGM), 8 to 38% affect the lateral head of the gastrocnemius, and 58 to 66% involve the soleus. 14 We believe that ultrasonographic studies of muscle architecture can support further research on the treatment and prevention of muscle injuries.

The objective of the current study was to compare the PA differences in the MGM in orthostasis and bilateral maximum plantar flexion (BMPF) in runners and non-runners.

## Materials and Methods

The Research Ethics Committee approved the present study under CAAE 02582718.0.0000.5505, opinion number 3.115.229. This study was clinical, observational, cross-sectional, and comparative.

The study included volunteers aged 18 to 60 years old. The non-runner group included subjects who had not practiced physical activity for over a year. The road runner group included those who ran for more than a year at least twice a week and five kilometers per week. We did not include subjects with musculoskeletal injuries, neuromuscular diseases, prostheses, acute or chronic pain in the lower extremities, cognitive disabilities, malignant diseases, or pregnancy.

The study participants completed a questionnaire, and their body mass index (BMI) was calculated as the ratio between weight (in kilograms) and the square of their height (in meters).

A physician specialized in musculoskeletal imaging and an orthopedist supervised the ultrasound examinations.

Images were obtained with a real-time ultrasound, model HD 11(Philips Medical Systems, Amsterdam, Netherlands), with a 9-MHz linear transducer and an acoustic coupling gel.

**Fig. 1:**
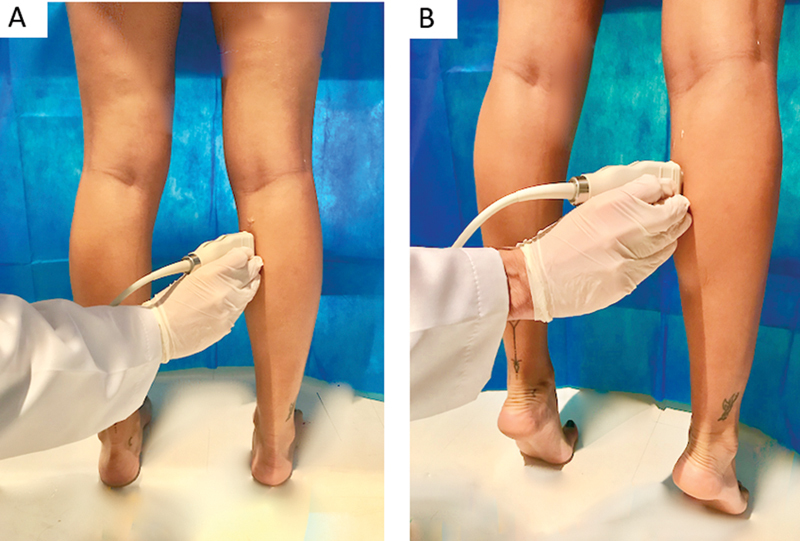
Orthostatic position ( **A** ) and maximum bilateral plantar flexion (BMPF) position ( **B** ) position of the medial gastrocnemius muscle.

To capture the image in the maximum cross-section area of MGM, the transducer was in a longitudinal position to the tibia, parallel to the muscle fascicles, and perpendicular to the skin in the upper third of an imaginary line from the flexion crease of the popliteal fossa to the center of the medial malleolus. 10 15 As described by Narici et al., 11 for the orthostatic examination, the volunteers remained in orthostasis with their feet parallel to the sagittal axis and the hip and knee in maximum extension ( Fig. 1A ).

**Fig. 2:**
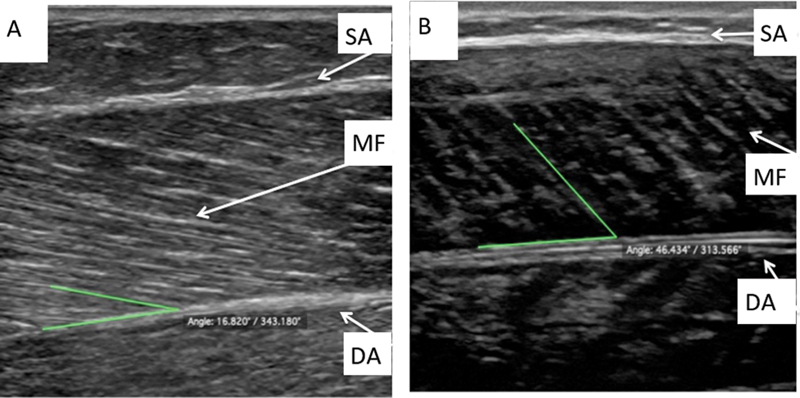
The arrows indicate a superficial aponeurosis (SA), a muscle fiber (MF), and a deep aponeurosis (DA). ( **A** ) Pennation angle (PA) of the medial gastrocnemius muscle in orthostasis. ( **B** ) Pennation angle of the medial gastrocnemius muscle during BMPF.

We asked the study participants to stand up and actively and gradually increase the force until BMPF for image capture on one leg ( Fig. 1B ), followed by a gradual relaxation for five seconds. Next, we instructed them to perform the same maneuver for image capture of the contralateral leg. Images were satisfactory when they allowed the delineation of multiple fascicles across the entire image without interruption. 10 16 Stored images underwent a subsequent analysis with the free open-source medical image viewer Horos (version 3.1.1) distributed under a Lesser General Public License (LGPL) at Horosproject.org. Pennation angle was the angle between the deep intramuscular aponeurosis and a visible muscle fascicle in orthostasis ( Fig. 2A ) and BMPF ( Fig. 2B ). 9 11 17 18 The same orthopedist obtained three PA measurements from each image, using different fibers. The data underwent statistical analysis.

### Statistical analysis

The Kolmogorov-Smirnov test assessed the normality of quantitative variables. The Student's t-test, Chi-squared test, and Pearson's correlation were used for data evaluation. The result of each comparison presented a *p* -value, defined as a statistical significance, at 0.05 (5%). Statistical analysis employed the following software: IBM SPSS Statistics for Windows, version 20.0 (IBM Corp., Armonk, NY, USA), Minitab version 16 (Minitab LLC, State College, PA, USA), and MS Excel 2010 (Microsoft Corp., Redmond, WA, USA).

## Results

The present study consisted of 62 volunteers, including 31 runners and 31 non-runners. The runner group had 14 (45.2%) females and 17 (54.8%) males. The non-runner group had 18 (58.1%) females and 13 (41.9%) males.

The runner group had 25 (80.6%) subjects with right-side dominance and 6 (19.4%) participants with left-side dominance. The non-runner group had 28 participants (90.3%) with right-side dominance and 3 (9.7%) with left-side dominance.

The age in the runner group ranged from 23 to 51 years, with a mean age of 35.6 years, and a coefficient of variation (CV) of 19%. The age in the non-runner group ranged from 18 to 59 years, with a mean value of 34.5 years, and a CV of 24%. According to the Chi-squared test, the groups were homogeneous regarding the dominant side, gender, and age.

The mean BMI was higher in the non-runner group (26.6) than in the runner group (22.40) ( *p* < 0.001).

Table 1 shows the descriptive statistical analysis of PA in orthostasis and BMPF of the right or left lower limbs from runners and non-runners.

**Table 1:** Pennation angle in the medial gastrocnemius muscle at ultrasound in road runners and non-runners

*Inferior limb*	*Condition*	*Group*	*PA (mean)*	*PA (median)*	*SD*	*CV*	*Min.*	*Max.*	*N*	*95%CI*	*p*
*Right*	*Orthostasis*	*Runners*	19.46	19.12	3.57	18	11.36	26.79	31	10.26	**00.004**
		*Non-runners*	22.25	22.04	3.77	17	13.23	30.23	31	10.33	
	*BMPF*	*Runners*	40.06	39.44	7.78	19	26.10	55.72	31	20.74	00.680
		*Non-runners*	40.89	40.47	8.07	20	25.42	56.25	31	20.84	
*Left*	*Orthostasis*	*Runners*	20.79	20.88	3.58	17	14.75	28.04	31	10.26	**00.029**
		*Non-runners*	22.83	22.45	3.61	16	15.12	29.45	31	10.27	
	*BMPF*	*Runners*	40.01	39.99	7.48	19	24.54	56.75	31	20.63	00.774
		*Non-runners*	40.52	39.95	6.38	16	27.56	59.26	31	20.25	

**Abbreviations:** PA, pennation angle; CV, coefficient of variation (in percentage); SD, standard deviation; BMPF, bilateral maximum plantar flexion; 95%CI, 95% confidence interval; Max., maximum; Min., minimum.

The Pearson's correlation was used to assess the correlation between MGM in orthostasis and BMPF of each leg in each group ( Table 2 ). We found a positive correlation (higher than 0) in all comparisons, which was greater in the right lower limb of the non-runner group.

**Table 2:** Correlation between bilateral medial gastrocnemius muscle pennation angles in runners and non-runners

*Group*	*Inferior limb*	*r*	*p*
*Runners*	*Right*	34.4	0.058
	*Left*	37.8	**0.036**
*Non-runners*	*Right*	60.7	**0.001**
	*Left*	26.3	0.152

Table 3 shows the descriptive statistical analysis of MGM's PA in orthostasis and BMPF of both lower limbs and gender.

**Table 3:** Measurement of the pennation angle of the medial gastrocnemius muscle by ultrasound examination in volunteer runners according to laterality and gender

*Inferior limb*	*Condition*	*Gender*	*PA (mean)*	*PA (median)*	*SD*	*CV*	*Min.*	*Max.*	*N*	*95%CI*	*p*
*Right*	*Orthostasis*	*F*	17.94	17.33	3.92	22	11.36	26.79	14	2.05	**0.029**
		*M*	20.71	21.64	2.78	13	13.60	24.24	17	1.32	
	*BMPF*	*F*	37.00	36.73	7.02	19	27.20	53.49	14	3.68	**0.045**
		*M*	42.57	43.21	7.66	18	26.10	55.72	17	3.64	
*Left*	*Orthostasis*	*F*	19.49	19.42	2.74	14	14.75	24.40	14	1.43	0.067
		*M*	21.85	21.10	3.90	18	16.93	28.04	17	1.85	
	*BMPF*	*F*	38.28	38.23	6.71	18	26.07	49.16	14	3.51	0.247
		*M*	41.45	42.36	7.98	19	24.54	56.75	17	3.79	

**Abbreviations:** PA, pennation angle; CV, coefficient of variation (in percentage); SD, standard deviation; F, female; BMPF, bilateral maximum plantar flexion; 95%CI, 95% confidence interval; M, male; Max., maximum; Min., minimum.

## Discussion

Scientific research has demonstrated that muscle architecture, including PA, fascicle length, and fiber composition and arrangement, affects muscle functions. 17 18 19 20

The first authors to describe PA were Gans and de Vree, in 1987, 21 when studying the external adductor muscle of a lizard's jaw. In 1992, Henriksson-Larsen et al. 22 evaluated the vastus lateralis muscle PA of 15 female volunteers. This was the first in vivo assessment of contraction and relaxation using an ultrasound device. The authors photographed the images for later PA analysis, being the first to demonstrate the feasibility of in vivo measurements and observing that the fiber angle in the relaxed position was smaller than in contraction. These results showed that the methodology for PA measurement using ultrasound images had high reliability. Our study evaluated PA in road runners compared with non-runners. We did not find any studies with this comparison in the literature.

The ultrasound method for capturing PA measurements from the MGM effectively determined muscle architecture parameters. 23 Its advantages included low relative cost, easy handling, portability, tissue contrast, good reproducibility, no radiation exposure, and real-time image analysis. 11 18 24 25 26

Our sample consisted of 62 participants. Only four studies (Binzoni et al., 27 Kubo et al., 15 28 and Kawakami et al. 29 ) presented a larger sample than ours. However, unlike us, these authors did not evaluate the PA during BMPF.

Regarding gender, the current study had a slight female predominance (51.61%; 32 out of 62), but without statistical significance. A paper on injuries in amateur runners demonstrated a male predominance. 30

In the present study, the mean PA in males was higher compared with females, consistent with the findings of other authors. 16 28 29

The mean age in years was 35.6 in runners and 34.5 in non-runners, contrasting with other authors, such as 22, according to Chow et al., 16 28.3, per Maganaris et al., 12 24, per Manal et al., 31 and 38.8, per Narici et al. 11 The mean age in our study was higher than in some studies in the literature. Kubo et al. 15 noted a significant difference in PA between young and elderly subjects only in the vastus lateralis muscle of the thigh. The same authors described that MGM did not present a statistically significant value regarding PA and age. We did not analyze whether age was a factor in MGM's PA range.

There are different methods for assessing muscle strength, including isometric, isokinetic, variable resistance, and free weight tests. The selection of a strength testing method involves specificities, ease of acquisition, cost, and safety. 32 Although runners perform monopodial support during their practice, we measured the PA during BMPF with the volunteer's body weight, as it is safe and easy to perform and analyze the data. We did not find any PA measurements in the literature using this method.

When comparing our PA data in the standing position with the literature, 11 12 15 we noted a non-significant difference of one or two degrees. Comparing road runners and non-runners, we found statistically significant values in the standing position and the BMPF.

Pearson's correlation was used to measure the degree of variable interconnection. We found a positive correlation of 60.7% when analyzing contraction in orthostasis and BMPF, indicating that the greater the angle in orthostasis, the greater the muscle contraction, consistent with the literature. 10 11 12 31

The present study had some limitations, such as considering road runners' answers as reliable regarding the time spent running and the distance covered per week. In addition, some runners may perform other types of physical exercise, such as weight training, swimming, and ballet, potentially changing the leg muscles.

Another limiting factor of our study was measuring a single variable from the muscle architecture, the PA, hindering the comparison with other muscle components. Muscle architecture presents several parameters for analysis, such as muscle thickness, fiber length, physiological cross-sectional area, and PA, which impact a muscle's force production capacity. Although ultrasound quality depends on the operator and is subject to interpretation errors, measurements of muscle structure following a protocol performed by sonographers with different training and experience can achieve objective image measurements with few differences and little variation in results. 33

Considering the great plasticity of the muscular system and the ability of skeletal muscles to adapt to different stimuli, such as disuse or training types, studies to elucidate the mechanisms by which muscle tissues adapt to these changes are relevant. For instance, we know that children with cerebral palsy have spastic muscles. We can evaluate the PA in contractures after surgical treatment for muscle stretching. In tendon transfers, we can choose muscles with similar PAs. In muscle injuries, we can monitor healing with serial PA assessments.

In sports medicine, we can study training variability by evaluating the PA in structural and functional muscle changes, especially in the force production capacity. A meta-analysis of Brazilian studies investigating the prevalence and risk factors for injury in amateur road runners detected a higher prevalence of injuries in men, most frequently muscle injuries (27.9%, 95% confidence interval [CI] 18.2–40.1%). 34 Timmins et al. 35 described a PA increase in the injured biceps femoris compared with the unaffected side. These findings encourage studies to help treat and prevent injuries early in road runners and other athletes.

## Conclusion

Road runners presented a significantly lower PA in MGM than non-runners in orthostasis.
