# Supplementary material for: Assessment of the Pennation Angle of the Medial Gastrocnemius Muscle in Road Runners and Non-runners
Source: Rev Bras Ortop (Sao Paulo). 2025 Apr 15;60(1):s00451804493. doi: 10.1055/s-0045-1804493 (PMC12020621; doi:10.1055/s-0045-1804493)
Supplement: Related texts

# Research article: Avaliação do ângulo de penação do músculo gastrocnêmio medial em corredores de rua e não corredores

**Keywords:** atividade motora, comportamento sedentário, contração muscular, músculo esquelético, ultrassonografia

## Abstract

**Objetivo** Avaliar o ângulo de penação (AP) do músculo gastrocnêmio medial (MGM) em ortostatismo e flexão plantar máxima bilateral (FPMB) em corredores de rua e não corredores.

**Métodos** Foram avaliados 31 corredores e 31 não corredores, de ambos os gêneros, entre janeiro e abril de 2019. O AP do MGM foi mensurado em ambos os lados por meio de ultrassom durante ortostatismo e apoio em flexão plantar máxima bilateral.

**Resultados** Os grupos foram considerados homogêneos quanto ao lado dominante, gênero e idade. Durante o ortostatismo, os corredores apresentaram no lado direito um AP médio de 19,46° em comparação a 22,5° dos não corredores ( *p* < 0,004) e no lado esquerdo apresentaram um AP médio 20,79° para corredores versus 22,83° para não corredores ( *p* < 0,029). Durante a FPMB, os corredores apresentaram no lado direito um AP médio de 40,06° em comparação a 40,89° dos não corredores e no lado esquerdo apresentaram um AP médio 40,01° para corredores versus 40,52° para não corredores. O AP do MGM do membro direito em corredores do gênero masculino foi estatisticamente significante maior, tanto em ortostatismo ( *p* < 0,029) quanto em FPMB ( *p* < 0,045). A correlação entre ortostatismo e FPMB do MGM da perna em cada um dos grupos demonstrou ser estatisticamente significante no grupo de não corredores para o membro inferior direito.

**Conclusão** Os corredores de rua apresentaram um valor significativamente menor do AP do MGM em relação aos não corredores em ortostatismo.

## Introdução

A corrida se originou na pré-história, quando os homens precisavam se movimentar rapidamente para caçar ou fugir de seus predadores. A prática da corrida, mesmo que seja de 5 a 10 minutos por dia e em velocidades lentas (< 9,65 km/h), está associada a riscos significativamente reduzidos de morte por todas as causas e, especialmente, por doenças cardiovasculares. Este estudo pode motivar indivíduos saudáveis, mas sedentários, a começar e continuar a correr em busca de benefícios de mortalidade substanciais e alcançáveis. 1

A arquitetura muscular humana foi estudada em espécimes de cadáveres. Contudo, são escassos os dados disponíveis sobre músculos humanos vivos. 2 3 4 O tecido muscular esquelético apresenta propriedades que determinam suas características e desempenho, como a excitabilidade, contratilidade, extensibilidade e elasticidade. 5 As propriedades mecânicas do músculo esquelético determinam seu desempenho, tais como força, comprimento, velocidade, trabalho e potência. 6

O ângulo de penação (AP) da fibra muscular é definido como o ângulo entre o fascículo muscular e o tendão intramuscular, isto é, a inclinação vertical das fibras musculares do longo eixo músculo-tendíneo. 5 7 8 O AP da fibra é uma importante característica funcional do músculo e varia com a intensidade da contração e o comprimento da fibra muscular. 9 A contração muscular provoca o encurtamento das fibras, levando a um aumento do AP. 9 10 Para obter medições precisas do comprimento ou do AP de um fascículo muscular, utiliza-se do exame de ultrassom. 10 11 12

A dor muscular na região posterior do membro inferior é queixa frequente em corredores. 13 14 O gastrocnêmio medial envolve 58 a 65% de todos os casos de lesões musculares da panturrilha; a cabeça lateral do gastrocnêmio, de 8 a 38% e o sóleo, de 58 a 66% dos casos. 14 Consideramos que o estudo da arquitetura muscular por ultrassom pode nos dar subsídios para futuros trabalhos sobre tratamento e prevenção dessas lesões musculares.

O objetivo deste estudo foi comparar as diferenças da mensuração do AP do músculo gastrocnêmio medial em ortostatismo e em flexão plantar máxima bilateral (FPMB) em corredores e não corredores.

## Materiais e Métodos

Este estudo foi previamente aprovado pelo Comitê de Ética em Pesquisa sob o número CAAE 02582718.0.0000.5505, parecer n° 3.115.229. O estudo foi clínico, observacional, transversal e comparativo.

Foram incluídos voluntários com idade superior a 18 e inferior a 60 anos. No grupo de não corredores foram incluídos indivíduos que não praticavam atividade física há mais de um ano. No grupo de corredores de rua, foram incluídos aqueles que praticavam a atividade esportiva de corrida há mais de um ano com frequência mínima de duas vezes por semana e mínimo de cinco quilômetros por semana. Não foram incluídos indivíduos com lesões musculoesqueléticas, com doenças neuromusculares, próteses, dor aguda ou crônica das extremidades inferiores, deficiências cognitivas, doenças malignas ou gravidez.

Os participantes do estudo preencheram um questionário e tiveram seu índice de massa corporal (IMC) calculado pela relação do peso (em quilos) com sua altura ao quadrado (em metros).

Os exames de ultrassonografia foram realizados sob supervisão de um médico especialista em imagem musculoesquelética e um ortopedista.

Obtiveram-se as imagens com um ultrassom, modelo HD 11, em tempo real (Philips Medical Systems, Amsterdã, Holanda) com um transdutor linear de frequência 9 mHz revestido com gel de transmissão para obter acoplamento acústico.

Para captura da imagem na área de secção transversal máxima do músculo gastrocnêmio medial, o transdutor foi colocado em posição longitudinal em relação à tíbia, orientado paralelamente aos fascículos musculares e perpendicular à pele, no terço superior de uma linha imaginária da prega de flexão da fossa poplítea até o centro do maléolo medial. 10 15 Conforme descrito por Narici et al., 11 para o exame em ortostatismo, os voluntários ficaram na posição ortostática, com os pés paralelos ao eixo sagital, com o quadril e joelho em extensão máxima ( Fig. 1A ).

**Fig. 1:** Posição de ortostatismo ( **A** ) e posição de flexão plantar máxima bilateral (FPMB) ( **B** ) do músculo gastrocnêmio medial.

A partir do ortostatismo, os participantes do estudo foram instruídos a desenvolver ativamente uma força gradualmente crescente até a FPMB para a captura da imagem em uma perna ( Fig. 1B ), seguido por um relaxamento gradual de cinco segundos. Em seguida, eram orientados verbalmente para a mesma manobra com a finalidade de armazenar as imagens do gastrocnêmio medial da perna contralateral. As imagens foram consideradas satisfatórias quando vários fascículos puderam ser delineados em toda a imagem sem interrupção. 10 16 As imagens armazenadas foram submetidas a análise subsequentes com um visualizador de imagens médicas de código aberto gratuito Horos (versão 3.1.1), distribuído sob Licença Pública Geral Menor ( *Lesser General Public License* – LGPL, em inglês) no Horosproject.org. O ângulo de penação foi calculado como aquele entre a aponeurose intramuscular profunda e um fascículo muscular claramente visível, tanto em ortostatismo ( Fig. 2A ) como durante a FPMB ( Fig. 2B ). 9 11 17 18 Foram realizadas, em cada imagem, três medições do AP, em fibras diferentes, pelo mesmo ortopedista. Os dados foram enviados para análise estatística.

**Fig. 2:** As setas indicam a aponeurose superficial (AS), uma fibra muscular (FM) e aponeurose profunda (AP). ( **A** ) Ângulo de penação (AP) do músculo gastrocnêmio medial em ortostatismo. ( **B** ) Ângulo de penação do músculo gastrocnêmio medial durante a FPMB.

## Análise estatística

Para avaliar a normalidade das variáveis quantitativas, o teste de Kolmogorov-Smirnov foi empregado. Os dados foram avaliados por meio dos testes T de Student, Qui-Quadrado e correlação de Pearson. O resultado de cada comparação apresenta um valor *p* , sendo definido como um valor de significância de 0,05 (5%). Para a análise estatística foram utilizados os softwares: IBM SPSS Statistics for Windows (IBM Corp., Armonk, NY, EUA), versão 20.0, Minitab versão 16 (Minitab LLC, State College, PA, EUA) e MS Excel 2010 (Microsoft Corp., Redmond, WA, EUA).

## Resultados

Sessenta e dois voluntários participaram do estudo, sendo 31 corredores e 31 não corredores. O grupo de corredores de rua foi formado por 14 (45,2%) indivíduos do gênero feminino e 17 (54,8%) do masculino. O grupo de não corredores tinha 18 (58,1%) indivíduos do gênero feminino e 13 (41,9%) do masculino.

No grupo de corredores, 25 (80,6%) participantes apresentavam dominância do lado direito e 6 (19,4%) participantes do lado esquerdo. No grupo de não corredores, 28 participantes (90,3%) apresentavam dominância do lado direito e 3 (9,7%) do lado esquerdo.

A idade no grupo de corredores variou de 23 a 51 anos, com média de 35,6 anos e coeficiente de variação (CV) de 19%. A idade no grupo de não corredores variou de 18 a 59 anos, com média de 34,5 anos e CV de 24%. Pelo teste do Qui-quadrado **,** os grupos foram homogêneos quanto ao lado dominante, gênero e idade.

Na comparação do IMC, a média foi maior no grupo de não corredores (26,6) do que no grupo de corredores (22,40) ( *p* < 0,001).

Na Tabela 1 , encontra-se análise estatística descritiva do AP em ortostatismo ou em FPMB dos membros inferiores direito ou esquerdo de corredores e não corredores.

**Tabela 1:** Mensuração do ângulo de penação do músculo gastrocnêmio medial durante exame de ultrassom em corredores de rua e não corredores

*Membro inferior*	*Condição*	*Grupo*	*AP (média)*	*AP (mediana)*	*DP*	*CV*	*Mín.*	*Máx.*	*N*	*IC95%*	*p*
*Direito*	*Ortostatismo*	*Corredores*	19,46	19,12	3,57	18	11,36	26,79	31	1,26	**0,004**
		*Não corredores*	22,25	22,04	3,77	17	13,23	30,23	31	1,33	
	*FPMB*	*Corredores*	40,06	39,44	7,78	19	26,10	55,72	31	2,74	0,680
		*Não corredores*	40,89	40,47	8,07	20	25,42	56,25	31	2,84	
*Esquerdo*	*Ortostatismo*	*Corredores*	20,79	20,88	3,58	17	14,75	28,04	31	1,26	**0,029**
		*Não corredores*	22,83	22,45	3,61	16	15,12	29,45	31	1,27	
	*FPMB*	*Corredores*	40,01	39,99	7,48	19	24,54	56,75	31	2,63	0,774
		*Não corredores*	40,52	39,95	6,38	16	27,56	59,26	31	2,25	

**Abreviaturas:** AP, ângulo de penação; CV, coeficiente de variação (em porcentagem); DP, desvio padrão; FPMB, flexão plantar máxima bilateral; IC95%, intervalo de confiança de 95%; Máx., máximo; Mín., mínimo.

Para correlação entre ortostatismo e FPMB do músculo gastrocnêmio medial da perna em cada um dos grupos, utilizamos a correlação de Pearson ( Tabela 2 ). Encontramos uma correlação positiva (maior que 0) em todas as comparações, sendo maior no grupo não corredores para o membro inferior direito.

**Tabela 2:** Correlação entre os ângulos de penação do músculo gastrocnêmio medial bilaterais em corredores e não corredores

*Grupo*	*Membro inferior*	*r*	*p*
*Corredores*	*Direito*	34,4	0,058
	*Esquerdo*	37,8	**0,036**
*Não corredores*	*Direito*	60,7	**0,001**
	*Esquerdo*	26,3	0,152

Na Tabela 3 , encontra-se análise estatística descritiva do AP do músculo gastrocnêmio medial em ortostatismo e FPMB de ambos os membros inferiores detalhadas conforme o gênero dos corredores.

**Tabela 3:** Mensuração do ângulo de penação do músculo gastrocnêmio medial pelo exame de ultrassom em corredores de acordo com a lateralidade e o gênero

*Membro inferior*	*Condição*	*Gênero*	*AP (média)*	*AP (mediana)*	*DP*	*CV*	*Mín.*	*Máx.*	*N*	*IC95%*	*p*
*Direita*	*Ortostatismo*	*F*	17,94	17,33	3,92	22	11,36	26,79	14	2,05	**0,029**
		*M*	20,71	21,64	2,78	13	13,60	24,24	17	1,32	
	*FPMB*	*F*	37,00	36,73	7,02	19	27,20	53,49	14	3,68	**0,045**
		*M*	42,57	43,21	7,66	18	26,10	55,72	17	3,64	
*Esquerda*	*Ortostatismo*	*F*	19,49	19,42	2,74	14	14,75	24,40	14	1,43	0,067
		*M*	21,85	21,10	3,90	18	16,93	28,04	17	1,85	
	*FPMB*	*F*	38,28	38,23	6,71	18	26,07	49,16	14	3,51	0,247
		*M*	41,45	42,36	7,98	19	24,54	56,75	17	3,79	

**Abreviaturas:** AP, ângulo de penação, CV, coeficiente de variação (em porcentagem); DP, desvio padrão; F, feminino; FPMB, flexão plantar máxima bilateral; IC95%, intervalo de confiança de 95%; M, masculino; Máx., máximo; Mín., mínimo.

## Discussão

Pesquisas científicas têm demonstrado que as funções musculares são afetadas pelas características da arquitetura muscular, tais como o AP, o comprimento do fascículo, a composição e o arranjo das fibras musculares. 17 18 19 20

O AP foi descrito pela primeira vez em 1987 por Gans e de Vree 21 por meio de um estudo do músculo adutor externo da mandíbula de um lagarto. Em 1992, Henriksson-Larsen et al. 22 avaliaram o AP do músculo vasto lateral da coxa de 15 voluntárias do sexo feminino. O músculo foi avaliado pela primeira vez *in vivo,* nas posições relaxado e contraído, com o uso do aparelho de ultrassonografia. Os autores fotografaram as imagens para posterior análise do AP, sendo os primeiros a demonstrar que essas medidas são possíveis *in vivo* e observando que o ângulo da fibra na posição relaxado era menor do que na posição em contração muscular. Os resultados obtidos demonstraram que a metodologia utilizada para medição do AP realizada em imagens de ultrassom apresentou alta confiabilidade. Nosso estudo avaliou o AP em corredores de rua em comparação com não corredores. Não encontramos na literatura trabalhos com essa comparação.

O método de ultrassonografia para captar as medidas do AP do músculo gastrocnêmio medial foi considerado eficaz para a determinação de parâmetros da arquitetura muscular. 23 Apresenta vantagens, como menor custo relativo, fácil manuseio e portabilidade, contraste entre os tecidos, boa reprodutibilidade, não exposição do indivíduo à radiação e possibilidade de análise da imagem em tempo real. 11 18 24 25 26

Nossa amostra foi composta de 62 participantes. Somente quatro estudos (Binzoni et al., 27 Kubo et al. 15 28 e Kawakami et al. 29 ) apresentaram uma amostra maior do que a nossa. Contudo, esses autores não avaliaram o valor do AP durante a FPMB, como realizado em nosso trabalho.

Neste estudo, em relação ao gênero, houve discreto predomínio do feminino, 51,61% (32 de um total de 62), porém sem apresentar significância estatística. Um estudo sobre lesões em praticantes amadores de corrida demonstrou predomínio em atletas do sexo masculino. 30

Neste estudo, a média do AP do gênero masculino foi maior do que a do feminino, corroborando com os achados de outros estudos. 16 28 29

Quanto à média de idade em anos, a do grupo dos corredores foi de 35,6 e a dos não corredores, de 34,5, sendo diferentes das reportadas por outros autores: 22 anos de média no estudo de Chow et al. 16 ; 28,3 anos no estudo de Maganaris et al. 12 ; 24 anos a média de idade no estudo de Manal et al. 31 ; e 38,8 anos no Narici et al. 11 Observamos que a média de idade do nosso estudo foi maior do que em alguns estudos da literatura. Kubo et al. 15 observaram uma diferença significativa no AP entre jovens e idosos somente no músculo vasto lateral da coxa. Os mesmos autores descreveram que o músculo gastrocnêmio medial não apresentava um valor estatisticamente significante em relação ao AP quanto à idade. Em nosso trabalho, não estudamos se a idade era um fator de variação nos valores do AP do músculo gastrocnêmio medial.

Para avaliar a força muscular, existem diferentes métodos, como o teste isométrico, o isocinético, o de resistência variável e o teste com pesos livres. A escolha de um método de teste de força envolve a especificidades, facilidade de aquisição, custo e segurança. 32 Embora os corredores realizem apoio monopodial durante sua prática esportiva, optamos por realizar a medição durante a FPMB com o peso corporal do voluntário, por ser seguro, de fácil execução e análise dos dados. Não encontramos na literatura medição do AP usando este método.

Quando comparamos nossos dados do AP em ortostatismo com os dados da literatura, 11 12 15 podemos observar que há uma diferença de um ou dois graus, não apresentando um valor significativamente diferente. Ao compararmos os grupos de corredores de rua e não corredores, encontramos valores estatisticamente significantes em ortostatismo e na FPMB.

Utilizamos a correlação de Pearson para mensurar o quanto as variáveis estão interligadas. Foi encontrada uma correlação positiva, com 60,7%, ao analisar o ortostatismo e a contração FPMB, indicando que, quanto maior o ângulo em ortostatismo, maior também será na contração muscular. Esses dados também estão de acordo com a literatura. 10 11 12 31

Este estudo possui algumas limitações, como termos que considerar fidedignas as respostas dos corredores de rua participantes do estudo, quanto ao tempo de corrida de rua e a quilometragem executada durante a semana. Devemos considerar também a possibilidade de que alguns corredores realizam outras modalidades de exercício físico, como musculação, natação, balé, entre outros. O que pode acarretar alterações na musculatura da perna.

Outro fator limitante de nosso estudo foi que medimos somente uma variável da arquitetura muscular, o AP, o que inviabiliza comparação entre os componentes musculares. A arquitetura muscular apresenta vários parâmetros para análise, como a espessura muscular, o comprimento da fibra, a área de secção fisiológica e o AP, que possuem um efeito na capacidade de produção de força de um músculo. Apesar da qualidade do ultrassom depender do operador e estar sujeita a erros interpretativos, medições da estrutura muscular seguindo um protocolo, realizado por ultrassonografistas com diferentes formações e experiências, podem conseguir uma medição objetiva de imagens com poucas diferenças e pouca variação nos resultados. 33

Considerando a grande plasticidade do sistema muscular, e a capacidades dos músculos esqueléticos de se adaptarem a diferentes estímulos, tais como o desuso ou diferentes tipos de treinamentos, assim, torna-se relevantes conduzir estudos para elucidar os mecanismos pelos quais os tecidos musculares se adaptam a essas modificações. Por exemplo, sabemos que em crianças com paralisia cerebral a musculatura se torna espástica. Podemos avaliar o AP nas contraturas após o tratamento cirúrgico para alongamentos musculares. Nas transferências tendíneas, podemos escolher músculos com APs semelhantes. Em se tratando de lesões musculares, podemos acompanhar a cicatrização dessas lesões com avaliações seriadas dos APs.

Na medicina esportiva, pode-se estudar a variabilidade de treinamentos avaliando o AP nas alterações estruturais e funcionais nos músculos, principalmente na capacidade de produção de força. Em uma metanálise de estudos brasileiros para investigar a prevalência e os fatores de risco de lesão em corredores de rua amadores, observou-se maior prevalência de lesões em homens, e as lesões musculares foram as mais frequentes (27,9%, intervalo de confiança [IC] de 95% 18,2–40,1%). 34 Timmins et al. 35 descreveram o aumento do AP em bíceps femurais lesionados em comparação ao lado não lesionado. Esses achados estimulam a realizar estudos com o intuito de auxiliar a tratar e prevenir lesões de maneira precoce em corredores de rua e outros atletas.

## Conclusão

Os corredores de rua apresentaram um valor significativamente menor do AP do músculo gastrocnêmio medial em relação aos não corredores em ortostatismo.
